# How “Light” Is “Light Smoking”? On the Cognitive Power of Nicotine Dependence

**DOI:** 10.3390/bs14111075

**Published:** 2024-11-11

**Authors:** Paolo Enrico, Federico Zorzi, Rachele Fanari, Arcangelo Francesco Uccula, Beniamina Mercante

**Affiliations:** 1 Department of Biomedical Sciences, University of Sassari, 07100 Sassari, Italy; enrico@uniss.it; 2 Department of Pedagogy, Psychology, Philosophy, University of Cagliari, 09127 Cagliari, Italy; federico.zorzi@unica.it (F.Z.); rfanari@unica.it (R.F.); 3 Department of History, Human Sciences and Education, University of Sassari, 07100 Sassari, Italy; uccula@uniss.it; 4 Institute of Biophysics, National Research Council, 90146 Palermo, Italy

**Keywords:** nicotine dependence, light smoking, attachment style, emotional dysregulation

## Abstract

In recent years, habits related to smoking have been changing. An increasing portion of light/occasional smokers tend to define themselves as non-smokers, leading to an incorrect perception of the risks that smoking even a few cigarettes can entail. In this study, we investigated the nicotine-induced cognitive distortion in young, higher-education students with low/moderate dependence (as indexed by the Fagerstrom questionnaire). The study involved 111 participants (62 female; mean age 24.43 ± 3.77) divided into smokers and non-smokers, who responded to specific questionnaires to evaluate their attachment style, emotion dysregulation, and state anxiety. Their response to smoking-related cues following emotional stimulation was experimentally evaluated, with participants being made to choose between care- or smoking-related images, following the presentation of threatening or neutral stimuli. The results show a cognitive bias in smokers, with participants choosing smoking-related stimuli significantly more often than non-smokers, with a slower reaction time, regardless of emotional cues. Emotion dysregulation and attachment style were also significantly correlated with response choice but not with response latency. Overall, our data indicate that there is no such thing as light use of nicotine and that smoking, even if not continuous, determines cognitive biases that lead to a vision of the environment as a function of substance seeking.

## 1. Introduction

Tobacco is one of the most widespread psychoactive drugs worldwide, with an estimated 1.245 billion users of one or more tobacco products in 2022 (WHO data). Such a large use of tobacco is due to different reasons, the first and foremost being nicotine (NIC), the main psychoactive alkaloid of *Nicotiana tabacum*, whose reinforcing effects are largely acknowledged to be the primary mechanism for both the initiation and continuation of tobacco use [1,2].

NIC’s effects on the central nervous system (CNS) are mediated by its agonist activity on the nicotinic subtype of acetylcholine receptors (nAChRs) [3,4]. Broadly distributed in the CNS, nAChRs are primarily located at the presynaptic level [1,2], and their activation stimulates the release of various neurotransmitters, such as glutamate, gamma aminobutyric acid, norepinephrine, and dopamine [5]. Relevant evidence shows that nAChR activation can significantly impact brain functions, enhancing attention, learning, and memory [6,7,8].

Tobacco smoke is a major public health concern, being the greatest avoidable health threat [6,7,8] and killing over 8 million people worldwide [9]. Due to the efforts of anti-smoking campaigns, the number of individuals dependent on tobacco is expected to continue decreasing in the future, especially in Western countries. However, over the past two decades, the modality of tobacco smoking has changed, with a higher percentage of light and/or intermittent smokers compared to heavy smokers [10,11,12]. This phenomenon may be due to different factors, including, possibly, a wrong perception of the risks and consequences of smoking “just a few cigarettes” [13,14,15]. While consistent evidence shows that heavy smokers (more than twenty-five cigarettes per day) are clearly dependent on cigarettes, studies on light/intermittent smokers (one to ten cigarettes per day) are still yielding unclear results [16,17,18]. This may be due to several reasons, but may also be because light smokers often do not view themselves as “real smokers”, causing the underestimation of their number and reinforcing the belief that light smoking does not pose significant risks. Despite this, it has been shown that being a light smoker does not indicate a higher likelihood of success in a cessation program [19], suggesting that the addictive effects of NIC may be more relevant than perceived.

The notion of addiction as a brain disease is largely accepted in the neuroscience community [20]. This view posits that addiction is a chronic, relapsing brain disorder marked by compulsive drug seeking and use, sustained by functional changes to brain circuits related to reward and self-control [20]. Abused drugs such as NIC are thought to hijack the reward network, ultimately promoting drug intake by a mechanism known as incentive salience, a cognitive process that motivates behavior toward rewarding stimuli [21,22]. Consequently, any smoking-related signal becomes highly attractive, hard to ignore, and can drive the NIC-dependent subject’s attention toward it [21,22].

Personality and social factors are also not extraneous to the development and maintenance of NIC dependence [23,24,25]. Specifically, consistent evidence indicates that smokers, in comparison to non-smokers, tend to be more impulsive, depressive, and anxious [23,24,25]. Substance use can also be associated with emotion regulation strategies, which are defined as the cognitive and behavioral processes shaping the affective states or the expression of emotions [26,27,28]. There is also evidence of an association between smokers’ emotion regulation difficulties and attentional bias to smoking-related cues [29]. Addiction and substance use disorders have also been associated with the attachment style of individuals [30,31,32]. More in detail, while a secure attachment facilitates overcoming stressful situations, an insecure attachment is frequently considered a risk factor for many psychiatric disorders and dysfunctional behaviors [33]. Relevant evidence shows that an insecure attachment style can play a significant role in developing and maintaining a smoking habit, while a secure attachment may be a protective factor [34,35,36]. The available evidence clearly indicates that a smoker’s compulsive behavior is not solely driven by NIC withdrawal, but emerges from a complex interplay of factors, including physical NIC dependence, emotional regulation, and the use of smoking as a coping mechanism for an insecure attachment style.

Finally, education also plays a significant role in smoking behavior, with an inverse relationship between educational accomplishments and smoking prevalence [37,38,39]. Interestingly, light or intermittent smoking is reported to be common in the higher-education population [10,40,41].

Understanding the interplay and the relative weight of these intertwined factors is essential for the development of effective smoking cessation strategies, in particular for those young subjects who do not perceive themselves as “real smokers”. In this study, we investigated the extent of the cognitive effect of NIC dependence in higher-education, light-smoking, low-NIC-dependence young adults, compared to higher-education non-smokers, using an experimental design aimed at assessing the propensity to seek care under distressed conditions. More in detail, our protocol aimed to exploit the NIC-induced incentive salience toward smoking-related cues in a situation where the prioritization of care-seeking should be the main behavioral driver [32,42,43].

We also investigated the participants’ cognitive status across various social and emotional dimensions using a battery of selected psychological tests, aiming to determine whether reactivity to smoking-related cues manifests differently across distinct cognitive domains involved in emotion regulation. We hypothesized that when primed with threatening images, care-related images will be chosen across all groups significantly more often, as a means of regulating negative emotions. Given the low NIC dependence of our participants, we also hypothesized that when primed with neutral images, there would not be a significant difference in the experimental task responses (image chosen and response latency) between the smokers and the non-smokers. No differences were expected in the filler (all neutral images) trials. Moreover, we expected the smokers to report significantly greater difficulties with emotion regulation strategies, which in turn are expected to be associated, along with insecure attachment styles, with more frequent choices of smoking-related images.

## 2. Materials and Methods

### 2.1. Type of Study

Observational case–control. Questionnaires scoring was carried out by staff blinded to the experimental group of the subjects.

### 2.2. Participants

One hundred and eleven healthy volunteers were recruited, between March and June 2024, from the local university population (School of Medicine and School of Psychology). The subjects were recruited through advertisements displayed in the faculties or through student social groups. According to the study’s inclusion and exclusion criteria, none of the participants had a history of, or current signs/symptoms of, neurological or psychiatric diseases, nor did they have current or recent use of any psychoactive drugs or use electronic cigarettes. All participants were native Italian speakers and had normal or corrected-to-normal vision.

### 2.3. Procedure

Participants sat in a comfortable chair in a quiet, light-controlled room, positioned 70 cm from a computer screen during the experiment. Visual stimuli were displayed via PsychoPy3 2023.2 software [44] on a 17″ LCD monitor (Samsung, Suwon-si, Republic of Korea, 75 Hz refresh rate). Participants were directed to maintain their gaze at the center of the screen; prior to beginning the experiment, they were provided with the following instructions (originally in Italian, translated into English): “You will see neutral images as well as images that might evoke negative emotions. Then, you will need to choose one of the two subsequent images—the one that can best help you overcome the negative emotion from the previous image”. A dedicated response pad was used to record the selected image and response time (RT, defined as the duration from the presentation of the stimulus to the completion of the participant’s key press).

**Figure 1:**
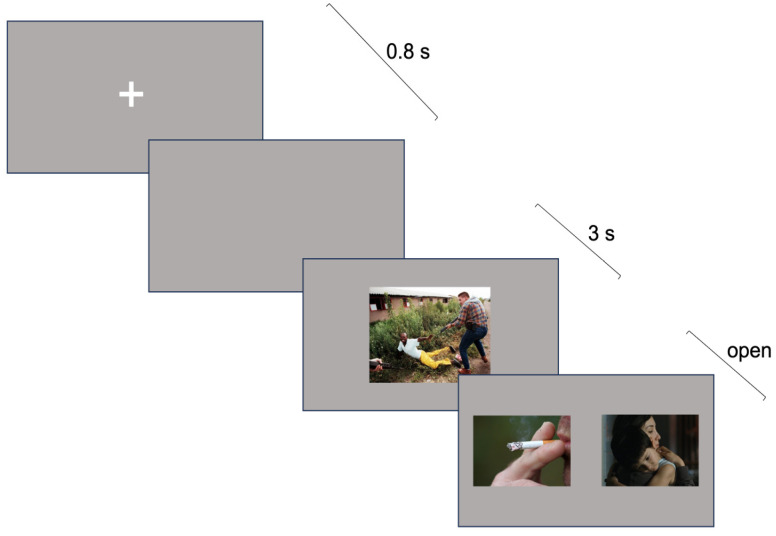
Depiction of the experimental task used in the study, illustrating the layout of visual stimuli over time.

During the experiment, participants were administered with sixty randomly intermixed trials: twenty with a threatening prime, twenty with a neutral prime, and twenty filler trials. Each trial consisted of four isoluminant, sequential images, starting with a fixation cross in the center of the screen (500 ms), followed by a gray screen (300 ms), then a prime picture (3 s), and finally two probe pictures presented side-by-side (Figure 1). The probe pictures remained on the screen until a response was made. In both the neutral and threatening conditions, one probe picture depicted a caring scenario (Care images) while the other depicted a smoking-related scenario (Smoking images); the relative position (left vs. right) of the probe pictures was counterbalanced among participants. In the filler trials, the probe pictures had neutral content. For each trial, the chosen image and the RT were recorded.

### 2.4. Measures

#### 2.4.1. Questionnaires

NIC dependence associated with cigarette smoking was assessed using the Italian version of the Fagerström Test for Nicotine Dependence (FTND; [45]). This self-administered questionnaire consists of 7 items designed to provide an ordinal measure of NIC dependence related to cigarette smoking by evaluating the quantity of cigarette consumption, the compulsion to smoke, and dependence. The FTND score ranges from 0 to 10 points and includes four categories: low dependence (0–2), medium dependence (3–4), strong dependence (5–6), and very strong dependence (7–10).

State anxiety was evaluated using the Italian version of the State–Trait Anxiety Inventory Form Y (STAI-Y) questionnaire [46,47]. The state anxiety form of the STAI-Y comprises 20 items rated on a 4-point Likert scale (from “Almost Never” to “Almost Always”), with higher scores reflecting greater state anxiety.

Emotion dysregulation was assessed with the Italian version of the Difficulties in Emotion Regulation Scale (DERS; [48,49]). The DERS is a self-administered questionnaire consisting of 36 items, each rated on a 5-point Likert scale ranging from 1 (“Almost never”) to 5 (“Almost always”). A total score can be calculated by summing all the items, along with scores for the following five subscales: Non-acceptance—a tendency to have a negative secondary or non-accepting reaction to one’s own distress; Goals—difficulty concentrating and/or accomplishing tasks when experiencing negative emotions; Impulse—difficulty remaining in control of one’s behavior when experiencing negative emotions; Awareness—lack of awareness or inattention to emotional responses; Strategies—a belief that there is little one can do to regulate oneself once upset; Clarity—the extent to which an individual knows and is clear about his or her emotions. Higher scores suggest greater difficulties in emotion regulation.

Attachment style was assessed using the Italian version of the Attachment Style Questionnaire (ASQ; [50]). This self-administered questionnaire designed to measure adult attachment comprises 40 statements, such as: “Overall, I am a worthwhile person”, “I am easier to get to know…”, and “I prefer to keep to myself…”. These statements are organized into five dimensions: Confidence (8 items), Discomfort with Closeness (10 items), Need for Approval (7 items), Preoccupation with Relationships (8 items), and Relationships as Secondary (7 items). Participants responded on a 6-point Likert scale, with anchor points ranging from 1 (totally disagree) to 6 (totally agree).

Participants completed the questionnaires and underwent the visual test in a random counterbalanced order: half began with the questionnaires and half with the test. Given that some of our young participants may have experienced stress in response to the experimental environment, we assessed state anxiety prior to the experiment to account for momentary anxiety and ensure the consistency of the results.

#### 2.4.2. Visual Stimuli

A total of hundred and eighty images were selected from the following databases to serve as priming or target images, as described in [51].

Priming images. Forty images selected from the International Affective Picture System (IAPS) which were consistently linked to higher or lower valence and arousal ratings were utilized to represent threatening (e.g., accidents, human attacks) or neutral (e.g., domestic objects) conditions [52]. Images for the threatening condition had mean valence ratings of 2.52 ± 0.64 and mean arousal ratings of 6.62 ± 0.41, while images for the neutral condition had mean valence ratings of 5.11 ± 0.32 and mean arousal ratings of 2.91 ± 0.68. Valence and arousal ratings showed significant differences between the two conditions, with F(1,38) = 264 for valence and F(1,38) = 438 for arousal.

Probe images. Forty images depicting comfort-related scenarios involving care were selected from the Besançon Affective Picture Set-Adult [53]. Two random sets of twenty images, each with consistent ratings of perceived comfort, valence, and arousal, were created: one for the threatening condition and one for the neutral condition. The two lists showed no significant differences in any dimension, with all F-values being less than 1. Forty pictures were taken from the SmoCuDa database [54]. These images depicted a wide range of smoking-related content and were randomly divided into two lists of twenty items each: one for the neutral condition and one for the threatening condition. The content was consistently balanced for valence and arousal ratings. The two lists did not significantly differ on any dimension, with arousal F(1,38) = 1.75, *p* = ns, and valence F(1,38) = 3.54, *p* = ns.

Sixty neutral pictures from the IAPS were chosen for the twenty filler trials.

### 2.5. Statistical Analyses

Sample size was determined a priori using the G*Power 3.1.9.4 program [55], ANOVA: repeated measures, within–between interaction (number of groups = 2, number of measurements = 3), alpha = 0.05, power (1 − β) = 0.95, effect size (Cohen’s f2) = 0.25. The power analysis resulted in n = 44 participants.

A repeated-measures Analysis of Variance (ANOVA) was performed to test the hypothesis of between-group differences (Smokers vs. NonSmokers) in the number of Smoking image choices (‘Ratio’) and in the response times (‘RT’) within the neutral-primed, the threatening-primed, and the neutral-filler experimental conditions. Then, a series of independent sample t-tests was computed to assess between-group differences in the STAI-Y, DERS, and ASQ scores. Within each group, one-tailed bivariate correlations were computed between the psychological and the ET measures. For the Smokers group, the correlations with age, duration of the habit, and FTND were calculated.

In case of a violation of the assumptions for the analyses, the corresponding non-parametric tests (Mann–Whitney U; Friedman’s ANOVA) and correlations (Kendall’s *τ*) were computed. Effect sizes were calculated as r coefficients, ranging from −1.0 to 1.0. Since we had directional hypotheses on the differences between both groups and conditions, all the tests performed had one-tailed *p*-value thresholds.

All the statistical analyses were performed using IBM SPSS v29.0.1.0.

### 2.6. Ethical Aspects

All experimental procedures were approved by the ethics committee of the University of Cagliari (UniCa, ID n. 0062572) and carried out in accordance with the Helsinki Declaration. No form of payment was given, and written informed consent was given by all participants prior to study entry.

## 3. Results

### 3.1. Sample Characteristics and Preliminary Analyses

The final sample was composed of 111 participants (62 females and 49 males; age: m = 24.43, SD = 3.77, range 19–32 years old). Participants were divided into two groups, based on their relationship with tobacco smoking: Smokers (n = 62; 29 females, age: m = 24.75 ± 4.2 years; 33 males, age: m = 24.33 ± 3.87 years); and NonSmokers (n = 49; 33 females, age: m = 24.42 ± 3.57 years; 16 males, age: m = 24.06 ± 3.47 years). Twenty-nine of the smoking participants (41.4%) reported a long-lasting smoking habit (6 or more years), with a median of 7 years (Min = 1, Max = 12); the reported mean cigarette consumption per day was 8 ± 3.2.

Since the variables were not normally distributed within the groups, and also violated the homogeneity of variance assumption, non-parametric tests were used in the subsequent analyses.

To support the reliability of the results, the possible effects of state anxiety on the experimental results were evaluated by comparing the scores of the two groups and by assessing their association with the behavioral measures. The results of the Mann–Whitney U test (see Section 3.5.1) showed no significant difference between the STAI-Y scores of Smokers and NonSmokers (U = 1452.0, *p* = 0.451). Moreover, two-tailed Kendall’s *τ* correlation coefficients between the STAI-Y scores, the Ratio, and the RTs were not statistically significant in either condition or group.

### 3.2. Group Comparisons of Behavioral Measures

#### 3.2.1. Choices

Two variables were computed to indicate the percentage of Smoking images chosen by the participants in the neutral-primed and in the threatening-primed conditions (respectively labeled the “Neutral-primed” to “Threatening-primed” Ratio). The Mann–Whitney U test showed that NonSmokers almost never chose Smoking images, while Smokers did it significantly more often in both conditions, with moderate effect sizes (Neutral-primed: RatioSmokers = 32.5%, RatioNonSmokers = 0.0%, U = 666.5, one-tailed *p* < 0.001; r = −0.50; threatening-primed: RatioSmokers = 25.0%, RatioNonSmokers = 0.0%, U = 874.5, one-tailed *p* < 0.001; r = −0.38).

Within the Smokers group, the Wilcoxon Signed-Rank tests also revealed that the difference between the two conditions was statistically significant, although with a small effect size (Z = −2.10, one-tailed *p* = 0.017, r = −0.19). As expected, no significant difference was found within the NonSmokers group (Z = −0.41, one-tailed *p* = 0.347, r = −0.04).

#### 3.2.2. Response Times

**Figure 2:**
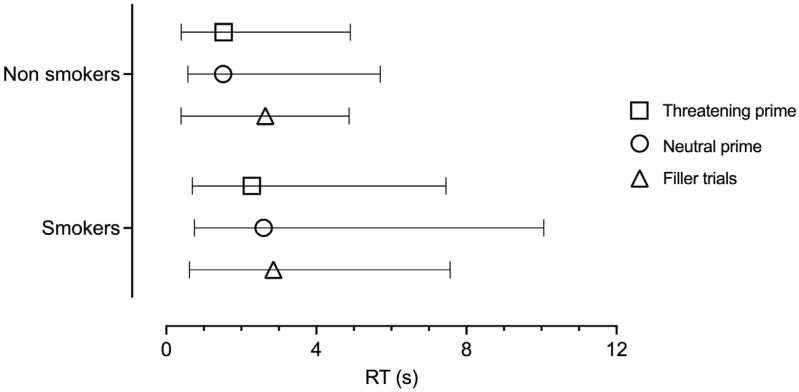
Smokers vs. NonSmokers: RT medians, minima, and maxima (in seconds) in the three conditions (threatening-primed, neutral-primed, and filler trials). Smokers had significantly slower RTs than NonSmokers in every condition (*p* < 0.001).

Between-group differences in the RTs in the three conditions (neutral-primed, threatening-primed, and neutral-fillers) were assessed with the non-parametric Mann–Whitney U test. The results showed that Smokers had significantly slower RTs in every condition, with the larger difference being within the neutral-primed one (neutral-primed: MdnSmokers = 2.59 s, MdnNonSmokers = 1.51 s, U = 805.0, one-tailed *p* < 0.001; r = −0.40; threatening-primed: MdnSmokers = 2.28 s, MdnNonSmokers = 1.52 s, U = 870.0, one-tailed *p* < 0.001; r = −0.37; neutral-fillers: MdnSmokers = 2.85 s, MdnNonSmokers = 2.64, U = 1124.0, one-tailed *p* = 0.009; r = −0.22; Figure 2).

The results of Friedman’s ANOVA showed that the RTs in the three conditions were significantly different within each group (Smokers: ꭕ2(2) = 17.16, *p* < 0.001; NonSmokers: ꭕ2(2) = 33.10, *p* < 0.001). Post hoc tests (Wilcoxon Signed-Rank test with Bonferroni corrected 0.016 level of significance) revealed that for the Smokers group, the threatening-primed RTs were significantly faster than those in the neutral-primed condition (Z = −3.34, one-tailed *p* < 0.001, r = −0.25) and slower than the neutral-fillers RTs (Z = −3.55, one-tailed *p* < 0.001, r = −0.26), while the neutral-primed RTs and the neutral-fillers RTs were not significantly different (Z = −1.83, one-tailed *p* = 0.033, r = −0.13). Within the NonSmokers group, the RTs in the threatening-primed condition were not significantly different from those in the neutral-primed condition (Z = −2.51, one-tailed *p* = 0.006, r = −0.21), while the RTs in the neutral-filler condition were significantly slower than both those in the neutral-primed (Z = −4.58, one-tailed *p* < 0.001, r = −0.38) and those in the threatening-primed condition (Z = −4.81, one-tailed *p* < 0.001, r = −0.40).

### 3.3. Relationships Between NIC Dependence Levels and RT

Twenty-nine of the smoking participants (41.4%) reported a long-lasting smoking habit (6 or more years), with a median of 7 years (Min = 1, Max = 20). The FTND results (Mdn = 3; Min = 0, Max = 8) indicated a low NIC dependence level for twenty-five participants (35.7%) and a mild level of dependence for fourteen participants (20%).

Kendall’s *τ* correlation coefficients (two-tailed) were computed between the age, the habit duration, the FTND, the Ratios, and the RTs in the experimental conditions. Predictably, the results showed a significant correlation of the habit duration with age (*τ* = 0.50, *p* < 0.001) and the FTND (*τ* = 0.28, *p* = 0.003). Also, the FTND result significantly correlated with the Ratios in both the neutral-primed (*τ* = 0.26, *p* = 0.005) and the threatening-primed (*τ* = 0.20, *p* = 0.033) conditions. In contrast, the RTs did not correlate with age, habit duration, or FTND in any experimental condition.

### 3.4. Psychological Constructs

A Mann–Whitney U test was performed to assess the difference between the scores of Smokers and NonSmokers on the psychological questionnaires. The results of the tests and the group descriptive statistics are reported in Table 1.

On the one hand, the results showed no significant difference in the attachment style (ASQ) measures (Confidence: *p* = 0.497; Discomfort with Closeness: *p* = 0.143; Relationships as Secondary: *p* = 0.072; Need for Approval: *p* = 0.382; Preoccupation with Relationships: *p* = 0.381). On the other hand, most of the emotion regulation scores were significantly higher in the Smokers group, although with relatively small effect sizes (i.e., DERS Goals: *p* = 0.026, r = −0.19; Awareness: *p* = 0.042, r = −0.17; Clarity: *p* = 0.015, r = −0.21; Total score: *p* = 0.020, r = −0.20).

### 3.5. Associations Between the Psychological Constructs and the RT Measures

Within each group, a series of Kendall’s *τ* correlation coefficients (one-tailed) were computed between the psychological measures, the Ratios (neutral-primed and threatening-primed), and the RTs (neutral-primed, threatening-primed, and neutral-fillers). Given the large number of comparisons, the Benjamini–Hochberg Procedure was applied to control the Type I error risk, with a false discovery rate of 20%, thus lowering the significant *p*-values to <0.05 and <0.01. The complete results are presented in Table 2 and Table 3 and summarized in the following paragraphs.

#### 3.5.1. Emotion Regulation Strategies (DERS)

In the Smokers group, the results showed that all but the NonAcceptance and the Impulsiveness DERS scores were significantly correlated with the neutral-primed Ratio (Goals: *τ* = 0.18, *p* < 0.05; Awareness: *τ* = 0.17, *p* < 0.05; Strategies: *τ* = 0.20, *p* < 0.05; Clarity: *τ* = 0.16, *p* < 0.05). Moreover, the DERS total score was also correlated with the threatening-primed Ratio (*τ* = 0.22, *p* < 0.01).

Conversely, the NonSmokers Impulsiveness scores were positively correlated with the neutral-primed Ratio (*τ* = 0.25, *p* < 0.05), as well as NonAcceptance (*τ* = 0.28, *p* < 0.01), which also significantly correlated with the threatening-primed Ratio (*τ* = 0.26, *p* < 0.05).

In both groups, none of the DERS scales were significantly correlated with the RTs in any experimental condition (Table 3).

#### 3.5.2. Attachment Style (ASQ)

The ASQ Confidence scores, expressing a more secure attachment style, were inversely correlated with the Ratios of the Smokers both in the neutral-primed (*τ* = −0.19, *p* < 0.05) and in the threatening-primed conditions (*τ* = −0.20, *p* < 0.05). Moreover, the latter was positively correlated with the scores of Relationships as Secondary, indicating a fearful/preoccupied attachment (*τ* = 0.21, *p* < 0.05) and Need for Approval, expressing a dismissing style (*τ* = 0.22, *p* < 0.01), which also correlated with the neutral-primed Ratios (*τ* = 0.19, *p* < 0.05).

Within the NonSmokers group, the Discomfort with Closeness scores (avoidant attachment style) were inversely correlated with the neutral-primed Ratios (*τ* = −0.19, *p* < 0.05). None of the other ASQ subscales were associated with the images chosen by the NonSmokers.

As with the DERS scores, the attachment style measures were not correlated with the RTs.

## 4. Discussion

In this study, we aimed to investigate the extent of the cognitive distortions induced by NIC dependence in a population of highly educated, light-smoking, low-NIC-dependence young adults, compared to a comparable group of non-smokers. To this end, we used a multidisciplinary approach combining a behavioral test with psychological measures, to account for the notion that both psychological and neurobiological factors are central to the disorder’s etiology.

Our behavioral data show that the cognitive influence exerted by NIC is clearly evident in the smoker group, implying that even minimal use of the substance can cause significant changes. We predicted that when primed with threatening images, participants in both groups will prioritize care-seeking in their choices, but that smokers will choose smoking-related images significantly more often than when primed with neutral images. This prediction is consistent with the observation that stressful events significantly impact the behavior of NIC-dependent subjects, leading to increased drug intake and a stronger urge to smoke [56,57]. While our data confirm the first prediction, we did not find significant differences in the selection of smoking-related images within the smoker groups following either neutral or threatening priming images. FTND values were also significantly correlated with the selection of smoking-related images in both the neutral- and the threatening-primed conditions. Thus, regardless of the type of prime used or the level of NIC dependence, smoking-related images were always able to attract the attention of the participants in the smoker group. This is also supported by the fact that non-smokers almost never selected smoking-related images.

Among the fundamental constructs that help to explain the behavior of addicted individuals is the notion of attentional bias (AB; [58,59]). This phenomenon refers to the tendency for substance-related stimuli to automatically and disproportionately capture or hold one’s attention, due to dysregulation of the brain’s motivational circuits induced by the substance used [60,61,62]. AB to smoking-related stimuli has been consistently observed in NIC dependents, and our data are in agreement with the literature, especially when considering the latency of the responses. We predicted that participants would respond faster when primed with threatening stimuli, with respect to neutral stimuli [63]. Our data are consistent with this hypothesis; however, we also found that smokers are significantly slower in their responses in both priming conditions compared to non-smokers. This observation may appear counterintuitive, since it has been shown that NIC addicts display faster reaction times to smoking-related cues [64,65,66]. However, our protocol was designed to highlight the NIC-induced cognitive distortion by creating a conflict when choosing between two possibly significant images, and the longer RTs measured when the smoking-related image was present are an epiphenomenon of this struggle. Smoking-related images bear no particular significance to non-smokers, and their shorter RTs express their ease of decision. This is also supported by the observation that in the filler trials, where all images are of neutral content, the RTs of Smokers and NonSmokers are comparable. Decision-making processes in addiction are consistently reported to be dysfunctional, leading to the inability to control behavior and the compulsion to seek and use the substance despite the consequences [67,68,69]. While the relationship between high levels of tobacco consumption and dependence is well documented in the literature, our data suggest that even small amounts of NIC can significantly impact decision-making processes. Lastly, we would like to highlight the interesting dissociation between the FNTD results and the behavioral data indicated by our observations.

Psychological constructs have a substantial influence on smoking behavior [23,70]. Since perceived stress levels can influence reactivity to stimuli [71], the possible effects of state anxiety (STAI-Y) on the experimental results were evaluated. As the results showed no significant differences between the two groups, or any association with the behavioral measures, we could therefore conclude that our measures were not significantly affected by the level of state anxiety.

Our results confirm the hypothesis that smokers had significantly greater difficulties in emotion regulation than non-smokers. More specifically, smokers had higher DERS total scores, as well as scores of the Goals, Awareness, and Clarity subscales. Further, within the Smokers group, the scores of the DERS Goals, Awareness, Strategies, and Clarity subscales (and DERS total score) were significantly associated with the neutral-primed choices, which is substantially consistent with previous research [26,72]. Interestingly, NonAcceptance and Impulsiveness were correlated with the choice of smoking-related images in the non-smoker group; this suggests that the miscues (as we considered the choice of a Smoking image by a non-smoker) could be influenced by emotion dysregulation, at least to some extent. This hypothesis should be further investigated within a specific research paradigm. Unexpectedly, none of the DERS scales were significantly correlated with the RTs in any experimental condition in both the smoker and non-smoker groups. This suggests that emotion dysregulation in smokers mainly influences the type of choice, rather than the cognitive effort behind it.

Consistently with the literature, the choices of the Smokers were also associated with their attachment style, as the ASQ Confidence scores (indicating a secure attachment) were inversely correlated with the Ratio of Smoking images chosen, while Relationships as Secondary (fearful/preoccupied attachment) and Need for Approval (dismissing attachment) scores were associated with higher Smoking image Ratios. These observations are consistent with the view that a secure attachment style is associated with a tendency to use internal methods for managing stress, possibly reducing the propensity toward the use of external regulation strategies [30,73]. Interestingly, the attachment style was also associated with the choices of the NonSmokers, as higher Discomfort with Closeness scores (avoidant attachment) were correlated with fewer choices of Care images. This result suggests that there could be an effect of the attachment style on miscues and attentional deployment, which should be further investigated. Similarly to the emotion regulation measures, none of the ASQ scores were associated with the RTs in any of the groups or conditions.

Limitations of the study and future directions. The main limitation of our study may be found in its key feature, namely the deliberate choice of a selected population of participants. In fact, owing to the known cognitive-enhancing effects of NIC, our population of smoking university students may be biased toward its use as a means to increase performances. A selection of participants from the general population may reveal further details. Further, our study addressed participants with low-to-moderate NIC dependence; studying high-dependence smokers would help generalize our results. In our protocol, we primed our participants with generic threatening images. Future studies may make use of images from anti-smoking campaigns to evaluate the effect of warnings that convey medical facts. Another key consideration is the relative novelty and descriptive focus of our work compared to prior research, which calls for caution when interpreting our results until more mechanistic results are available. Lastly, we did not include in our study subjects using other methods of NIC administration such as vaporizers or electronic cigarettes. Given the rapid and vast diffusion of these new methods and their large acceptance, especially among young adults, future studies addressing this subject are warranted.

## 5. Conclusions

Tobacco dependence will likely remain a relevant health problem in the near future, and in order to effectively address it, it is necessary to acknowledge that besides physical harm, smoking induces a variety of cognitive effects due to the interaction of pharmacological, psychological, and environmental factors. The use of reactive cue responses as an index of the extent of NIC-induced cognitive distortion may help us to understand the nature of attentional bias in smokers, as well as to characterize individual factors for the development of tailored interventions, with improved efficacy and tolerability.

Our data show that NIC use, even in small amounts and/or discontinuously, can induce significant cognitive distortions. This is particularly concerning when considering the characteristics of the study population; in fact, our participants, given their high socio-cultural background and field of study, should be aware of the risks associated with NIC dependence and thus refrain from smoking. Increasing education about NIC dependence, and its hidden cognitive effects, is thus particularly important when targeting young people who do not see themselves as “real smokers” due to their smoking habit. Further, smoking cessation programs should not underestimate the cognitive effects of even small and/or sporadic NIC intake, or the role of psychological and personality factors.

## Figures and Tables

**Table 1:** Between-group comparisons of the psychological measures.

	Valid n; Cronbach’s α	Smokers Median (Min:Max); Cronbach’s α	NonSmokers Median (Min:Max); Cronbach’s α	Mann–Whitney U	*p*-Value (1-Tailed)	Effect Size (r)
*Anxiety (STAI)*						
Total score	110; 0.90	44 (32; 52); 0.91	42 (33; 53); 0.88	1239.0	0.066	−0.14
*Emotion regulation (DERS)*						
NonAcceptance	109; 0.88	11 (6; 27); 0.89	12 (6; 25); 0.87	1373.5	0.279	−0.06
Goals *	110; 0.90	16 (5; 25); 0.92	12 (5; 25); 0.88	1172.0	0.026	−0.19
Impulsiveness	110; 0.89	10 (6; 26); 0.91	9 (6; 23); 0.81	1318.5	0.144	−0.10
Awareness *	110; 0.86	14 (6; 26); 0.87	12 (6; 23); 0.83	1207.0	0.042	−0.17
Strategies	110; 0.87	16 (8; 36); 0.87	14 (8; 30); 0.85	1311.0	0.135	−0.11
Clarity *	110; 0.83	11 (5; 24); 0.84	9 (5; 18); 0.78	1135.5	0.015	−0.21
Total score *	109; 0.94	77 (42; 150); 0.94	69 (38; 131); 0.93	1133.0	0.020	−0.20
*Attachment style (ASQ)*						
Confidence	111; 0.80	32 (17; 45); 0.82	32 (16; 43); 0.75	1517.5	0.497	0.00
Discomfort with Closeness	111; 0.78	39 (22; 56); 0.82	40 (24; 51); 0.70	1339.0	0.143	−0.10
Relationships as Secondary	111; 0.77	16 (7; 31); 0.71	13 (7; 30); 0.83	1273.0	0.072	−0.14
Need for Approval	111; 0.78	20 (9; 35); 0.77	20 (8; 39); 0.79	1468.0	0.382	−0.03
Preoccupation with Relationships	111; 0.69	28 (15; 41); 0.72	28 (18; 41); 0.64	1467.5	0.381	−0.03

* Difference is significant at *p* < 0.05 (one-tailed).

**Table 2:** Correlations between neutral-primed (N-P) and threatening-primed (T-P) Ratios and psychological constructs.

	Smokers		NonSmokers	
	N-P Ratio	T-P Ratio	N-P Ratio	T-P Ratio
*Anxiety (STAI)*				
Total score	0.13	0.10	−0.11	−0.07
*Emotion regulation (DERS)*				
NonAcceptance	0.06	0.07	0.28 **	0.26 *
Goals	0.18 *	0.12	0.16	0.11
Impulsiveness	0.07	0.15	0.25 *	0.16
Awareness	0.17 *	0.13	−0.05	−0.10
Strategies	0.20 *	0.14	0.12	0.09
Clarity	0.16 *	0.15	−0.03	−0.07
Total score	0.22 **	0.20 *	0.13	0.08
*Attachment style (ASQ)*				
Confidence	−0.19 *	−0.20 *	0.03	−0.02
Discomfort with Closeness	0.05	0.09	−0.19 *	−0.14
Relationships as Secondary	0.08	0.21 **	−0.01	−0.05
Need for Approval	0.19 *	0.22 **	−0.01	−0.01
Preoccupation with Relationships	0.11	0.10	−0.01	0.02

* correlation is significant at *p* < 0.05 (one-tailed). ** correlation is significant at *p* < 0.01 (one-tailed).

**Table 3:** Correlations between neutral-primed (N-P), threatening-primed (T-P), neutral-fillers (N-F), RTs (sec), and psychological constructs.

	Smokers			NonSmokers		
	N-P RT	T-P RT	N-F RT	N-P RT	T-P RT	N-F RT
*Anxiety (STAI)*						
Total score	0.03	0.03	−0.01	0.04	0.01	−0.05
*Emotion regulation (DERS)*						
NonAcceptance	0.01	−0.01	−0.10	0.12	0.10	0.01
Goals	0.02	0.01	−0.06	0.14	0.13	0.08
Impulsiveness	−0.06	−0.05	−0.14	0.09	0.10	0.09
Awareness	−0.11	−0.10	−0.14	−0.06	−0.05	−0.09
Strategies	0.14	0.11	0.04	0.04	0.05	0.01
Clarity	0.01	−0.01	0.01	−0.03	−0.07	−0.02
Total score	0.01	−0.01	−0.11	0.05	0.04	−0.01
*Attachment style (ASQ)*						
Confidence	−0.08	−0.11	−0.01	0.07	0.03	0.01
Discomfort with Closeness	−0.05	0.01	−0.12	−0.13	−0.09	−0.05
Relationships as Secondary	0.10	0.11	0.03	−0.11	−0.08	−0.05
Need for Approval	0.06	0.03	−0.04	0.01	0.02	−0.02
Preoccupation with Relationships	0.07	0.09	−0.06	0.04	0.03	−0.07

## Data Availability

Date available on request due to restriction.
